# Population health intervention research: what is the place for pilot studies?

**DOI:** 10.1186/s13063-019-3422-4

**Published:** 2019-05-30

**Authors:** Lehana Thabane, Linda Cambon, Louise Potvin, Jeanine Pommier, Joëlle Kivits, Laetitia Minary, Kareen Nour, Pierre Blaise, Julie Charlesworth, François Alla, Discussion Panel, François Alla, Pierre Arwidson, Pierre Blaise, Christopher Bonell, Isabelle Boutron, Linda Cambon, Rona Campbell, Patrizia Carrieri, Franck Chauvin, François Dabis, Nancy Edwards, Christine Ferron, Marie-Renée Guevel, Nadir Kellou, Joëlle Kivits, Antony Lacouture, Thierry Lang, Susan Michie, Laëtitia Minary, Graham Moore, Grégory Ninot, Kareen Nour, Jeanine Pommier, Louise Potvin, Lehana Thabane

**Affiliations:** 1 McMaster University, Hamilton, Canada; 2 Université de Bordeaux, Bordeaux, France; 3 Université de Montréal, Montreal, Canada; 4 European Center on Disease Prevention and Control, Stockholm, Sweden; 5 Université de Lorraine, Nancy, France; 6 ARS Pays de Loire, Nantes, France; 7 A Tree of Life Sciences Ltd, Manchester, UK

**Keywords:** Process evaluation, Complex intervention, Intervention research, Pilot studies

## Abstract

**Background:**

An international workshop on population health intervention research (PHIR) was organized to foster exchanges between experts from different disciplines and different fields.

**Aims:**

This paper aims to summarize the discussions around one of the issues addressed: the place or role of pilot studies in PHIR. Pilot studies are well-established in biomedical research, but the situation is more ambiguous for PHIR, in which a pilot study could refer to different purposes.

**Methods:**

The workshop included formal presentations of participants and moderated discussions. An oral synthesis was carried out by a rapporteur to validate by expert consensus the key points of the discussion and the recommendations. All discussions have been recorded and fully transcribed.

**Discussion:**

PHIR generally addresses complex interventions. Thus, numerous tasks may be required to inform the intervention and test different aspects of its design and implementation. While in clinical research the pilot study mainly concerns the preparation of the trial, in PHIR the pilot study focuses on the preparation of both the intervention and the trial. In particular, pilot studies in PHIR could be used for viability evaluation and theory development.

**Recommendations from the workshop participants:**

The following recommendations were generated by consensus from the workshop discussions: i) terms need to be clarified for PHIR; ii) reporting and publication should be standardized and transparency should be promoted; iii) the objectives and research questions should drive the methods used and be clearly stated; iv) a pilot study is generally needed for complex intervention evaluation and for research-designed programs; and v) for field-designed programs, it is important to integrate evaluability assessments as pilot studies .

**Conclusion:**

Pilot studies play an important role in intervention development and evaluation. In particular, they contribute to a better understanding of the mechanisms of intervention and the conditions of its applicability and transferability. Pilot studies could therefore facilitate evidence-based decisions about design and conduct of main studies aimed to generate evidence to inform public health policy.

## Introduction and problem statements

Population health intervention research (PHIR) can be defined as the use of scientific research methods to produce knowledge about policy and program interventions, whether or not they are conducted in the context of the health system/sector, and have the potential to make an impact at the population level [1]. There is a growing interest in PHIR at the present time; indeed, it has become a priority for health systems [2].

Population health interventions are generally, but not necessarily, considered as complex interventions, that is, “made up of various interconnecting parts” [3]. These interventions can also be considered as complex because of the influence of the context on their results [4]. The Medical Research Council Guidance provided recommendations to guide researchers in designing, developing, and evaluating complex health interventions, and more specifically process evaluation [5].

Despite progress and increasing interest in complex interventions since the MRC definitions in 2000, they continue to present significant challenges for research communities, practitioners, and decision-makers. In particular, issues relating to the transferability of results are critical because the environment and conditions of implementation are themselves determinants of the intervention outcomes [6].

While the MRC guidance on process evaluation [5] represents a key milestone, methods and tools to conduct evaluations need to be refined and there are many outstanding challenges and questions. There is a need not only to develop methods, tools, and practical guidance for researchers seeking to implement the latest MRC guidance, but also to clarify some underlying paradigms and to operationalize the overall research approach, from conceptualization to the dissemination of an intervention.

In France, where PHIR is well developed, the National Coordinated Action for Intervention Research (Action coordonnée pour la recherche interventionnelle en santé publique; ACRISP) was created in 2015 to support the development of research that is both scientifically accurate and useful to practitioners and policymakers; to promote the sharing of experience between researchers, practitioners, and policymakers; to encourage conceptual and methodological reflections; and to make proposals in terms of organization of research, regulation, and funding. In November 2016, ACRISP organized an international workshop bringing together some of the world’s leading experts and researchers. The objective was to promote exchanges between researchers from different disciplines due to the complexity of the field, which requires an interdisciplinary approach. The workshop provided an opportunity to share experiences and learning between researchers from various fields, such as clinical research, health services research, and population health intervention research. The invited researchers were particularly interested in methodological research (most of them had published methodological papers). Some of the key issues in PHIR were addressed. The presentations and discussions, in three successive sessions, covered various themes. One of them was the place of pilot studies in PHIR.

The term ‘pilot study’ is well-established in biomedical research and pilot studies for phase III trials are routinely performed in many areas of clinical research [7]. The situation is more ambiguous for PHIR, in which a pilot study could refer to different purposes. Yet, in this field, the complexity of the intervention, the necessary flexibility of its implementation in order to fit with the context, and the influence of its contextual parameters—individual, social, organizational, physical, etc.—require numerous preparatory tasks prior to the evaluation, influencing both the way the evaluation will be conducted and its results. Such tasks therefore have to be considered in pilot studies.

This article aims to share and synthesize the discussions, work, and recommendations put forward by experts during this workshop. Practically, the aim is to clarify what kind of pilot studies should be used in PHIR and for which objectives, through an account of the different questions raised and the consensual answers the experts provided. It is not intended to be a systematic synthesis of science or to bring new data, but to be a milestone for a common basis for discussion between researchers from different disciplines and fields.

## Methods

The workshop was prepared by LT and FA (preparation of a program and compilation of a bibliographic file). It included formal presentations of participants and discussions moderated by LT. At the end of the workshop, an oral synthesis was carried out by a rapporteur (JP) to validate by expert consensus the key points of the discussion and the recommendations. All the discussions and the validated synthesis were recorded. The recording has been fully transcribed. A first draft of the paper was prepared by JC and FA from this material, then corrected and validated by LT, then by all of the coauthors (who all participated in the debates).

## Discussion and key arguments

### What is a pilot and/or feasibility study?

There are many different definitions of pilot studies. These definitions have in common the concept of doing something on a smaller scale with the intent of gathering information to do something on a larger scale. However, a small-sized study is not necessarily in itself a pilot study. There are issues regarding language and terminology. Synonymous terms include feasibility study, vanguard study, pre-study, and first phase. Multiple terms are used without clear distinctions, e.g. pilot work, pilot study, feasibility study, pilot trial [8].

The terms pilot and feasibility study have often been used interchangeably [7]. There is no consensus on their definitions. Some people consider the two to be the same; others think they are different [9]. Some have used an overarching definition of feasibility studies, with pilot studies being a subset: “a feasibility study asks whether something can be done, should we proceed with it, and if so, how. A pilot study asks the same questions but also has a specific design feature: in a pilot study a future study, or part of a future study, is conducted on a smaller scale” [10].

Moreover, in current practice, there are questions about how to describe ‘preparatory trials’, i.e., the work done before starting a study. In some circumstances these may be covered by the general term feasibility assessment/trial. On the other hand, a phase that is in a study assessing uncertain aspects in order to refine the design, but is part of the same study, would be better described as an ‘internal pilot’ [11].

Pilot trials differ from princeps trials in their aims and objectives, focusing on assessing feasibility rather than effectiveness or efficacy. They are usually designed to support the development of a future definitive trial and generally provide a good way to assess the feasibility of the main trial. However, they are not usually well-described, the feasibility objectives are usually unclear, and the rationale for trialing feasibility is often missing. The analytical strategies on how to analyze feasibility outcomes lack clarity and feasibility criteria are often missing [12].

Many pilot studies are not published or even reported. This is sometimes because inappropriate theory produces negative results (pilot trials are not designed to produce statistical significance). They are sometimes not considered important enough to be published in the same way as the main trial. However, it has been suggested that they should be scrutinized in the same way as full trials and that they should require registration. Information should be publicly available—for those who participated and also to ensure that any mistakes are not repeated. We are aware that there are steps in place to improve this, as well as requirements imposed by funders to publish protocols and registration requirements prior to release of research funds.

It was noted that progress is being made to improve standardization and reporting of pilots. A CONSORT extension specifically for pilot trials is now available [12]. The development of this extension was motivated by the increasing number of studies described as feasibility or pilot studies and by research identifying weaknesses in their reporting and conduct. There is also a journal specifically for publishing pilot and feasibility studies (https://pilotfeasibilitystudies.biomedcentral.com/).

### Are the terms and methods for pilot studies in biomedical models also applicable to PHIR?

The types of models and approaches in the biomedical field may not be suitable for other types of interventions. It is useful at this point to try to see where terminologies might be appropriate and where they might not. The choice of these kinds of approach in PHIR requires an open mind and a more flexible outlook.

In particular, in PHIR complex interventions need to be addressed. Thus, before testing the evaluation, as in a traditional pilot or feasibility study, many tasks may be necessary to inform the intervention and test different aspects of its design and implementation. In other words, while in clinical research the pilot study mainly concerns the preparation of the trial, in PHIR a pilot study focuses on the preparation of both the intervention and the trial/evaluation.

### Which pilot study for field-designed programs?

We must distinguish programs designed and implemented by researchers from those built by the actors and decision-makers. In this latter case, researchers do not intervene but observe (i.e., natural experimental designs). The consensus is that a pilot study is generally needed for complex intervention evaluation for de novo research-designed programs. However, the place of a pilot study in real-life programs is less clear. In these cases, the concept of pilot study as defined in clinical research does not make any sense and the goal is more to ensure the evaluability of an ongoing program than the feasibility of research.

The concept of evaluability assessment developed by evaluators over the past 30 years can provide useful information on the role of pilot studies in these kinds of PHIR. Indeed, in 1980, Rutman proposed evaluability assessment [13], which can now be considered to essentially cover ideas addressing program relevance and questions such as the relevancy of the program according to targets, the existing theory of change, the responsivity of the implementation to local conditions, the nature of activities, the influence of the context on the results, etc. A report on publications of evaluability assessment commissioned by the Department for International Development in the UK [14] concluded that an evaluability assessment addresses what can be learnt from studying a program, in principle and in practice. Is the program based on known and tested theory? In practice, is it worth it and can it be done? In practice, evaluators have to describe the objectives, logic, and activities of the program with the aim of investigating its credibility, feasibility, sustainability, and acceptability.

Moreover, contrary to research-designed programs, field-designed programs have a genealogy: a previous version of this program was implemented before. In real life, the genealogy of programs may be traced through their previous iterations.

Therefore, this evaluability improves the understanding of how the intervention is set in context and refines the expected outcomes to be assessed in the efficacy study. It could constitute a subject for a pilot study in order to improve the program and prepare the efficacy study.

#### Which pilot study for research-based interventions?

In research-based intervention, there are many reasons to conduct pilot work in PHIR, including both evaluation and intervention aspects.

Similar to clinical research, a pilot study could assess the feasibility of procedures and methods, recruiting for a main trial and retaining subjects in a trial, collecting complete data, etc. Pilot work may also be used to gather preliminary feedback on an intervention and what might be refined for the main trial going forward, and also to measure adherence to protocol.

However, in complex interventions other key objectives could be considered.

Chen [15] recommends addressing the “viability validity” of an intervention as the first step in an evaluation process. He has defined this as “the extent to which an evaluation provides evidence that an intervention is successful in the real world”. This refers to the practical, affordable, suitable, evaluable, and helpful aspects of an intervention in the real world. So, it actually refers to the way an intervention fits with its context. In the viability study, the aim is to answer these questions: “Can it recruit and/or retain ordinary clients? Can it be adequately implemented by ordinary implementers? Is it suitable for ordinary implementing organizations to coordinate intervention-related activities? Is it affordable, is it evaluable and does it enable ordinary clients and other stakeholders to view and experience how well it solves the problem?” [15]. This viability study could take place within pilot studies to explore and understand how the intervention fits with the context. According to Chen, without a viable evaluation, we risk addressing the efficacy of an ‘off ground’ intervention that cannot be transferred to another context or scaled up. Overall, he has proposed to use an integrative validity approach, by firstly proceeding to a viability study, secondly carrying out an effectiveness study, taking into account the real conditions of implementation, and lastly (and if necessary) performing an efficacy study or directly proceeding to dissemination.

Moreover, like an evaluability assessment, a pilot study could also concern the reasons for the intervention in order to help to define the expected results. It could contribute to informing the design of the best intervention to fit the context, according to the best evidence on what works and how, along with contextual parameters, as recommended in theory-based intervention [15, 16]. Indeed, according to the intervention-based paradigm, all interventions are based on implicit or explicit theories. The principle of theory-based intervention is to make explicit what underlies the intervention: which activities, and under which conditions, produce which mechanisms of change, in turn producing the expected results. Making this underlying theory explicit is necessary to ensure that the intervention delivered is relevant to the production of the expected results. There are different methods to produce a theory-based intervention, but all of them cross scientific evidence, theoretical works, and stakeholders’ expertise in a participative and multidisciplinary way. This work could be an integral part of a pilot study.

Hence, in the framework of research-based intervention, a pilot study could assess the feasibility of the trial, but also contribute to the design of the theory-based intervention and assess its conditions of viability in the context. Knowledge would be provided on what will be evaluated (through a comprehensive explanation of what the intervention attempts to change by a theory-based approach), how the intervention has to be conducted in order to fit with the context and to be transferable (with the viability study), and how to conduct the effectiveness study (with the classic pilot study as in clinical research). A pilot study pursuing all of these objectives (feasibility, expected outcomes, investigation, and contextual factors) could enable practitioners to hypothesize what may be potentially effective in the context and under which conditions. Once this has been established, it is relevant to test the ‘real’ efficacy and effectiveness in the specific real world in which the intervention is to be implemented. This could be likened to phases I/II in drug development in the clinical field, whereby an intervention is tailored/adapted and refined to optimize its success in the main trial.

To conclude the discussion*,* from an intervention perspective in public health programs, pilots could be used in many ways: to test the intervention’s activity or strategy, to identify its most important components, to test ways of operating/administrating an intervention, or to explore how intervention protocols are followed by practitioners and clients. Pilot studies might be also the preferred option when they can focus on an in-depth evaluation of a specific aspect of an innovation. Moreover, pilot studies could provide a practical option if the funding for a randomized controlled trial is not available. Indeed, the proposal of a pilot may be helpful in negotiations with funding agencies and may enable a more rapid start, which could be followed by a quick transition to the full phase. Another advantage is that generally a full PHIR can last years. However, policymakers generally want answers quickly. A well-conducted pilot study could provide a quick answer that is useful for decision-making. This answer may lead, for example, to the decision of whether or not to implement the intervention, and how to implement it. Full research can then be done concurrently with the deployment of the intervention. Thus, in PHIR, a pilot study does not delay the decision but may advance it. Finally, in pilot studies in biomedical research, there are criteria to indicate whether to proceed with the full trial. The guiding principles for establishing the criteria to decide when to progress to the main study in PHIR are yet to be defined.

## Position statements, further research directions, and recommendations

Considering these discussions, the experts at the international workshop organized by ACRISP defined five recommendations for pilot studies in PHIR.

### Recommendation 1: Clarifying the terms for PHIR

In practice, different researchers appear to have preferences for different terms. It is therefore important that the terminology is consistent and understandable by all. It is not always possible or even desirable to have a strict definition of words. However, terminology should at least be explicitly stated at some stage, especially in a multidisciplinary field such as PHIR.

This clarification is a sine qua non condition to encourage, advocate, and train in the use of pilot studies in this field, more relevant in PHIR than in others because of the complexity of interventions.

### Recommendation 2: Standardizing reporting and publication

The need for high standards in conduct and reporting is just as important for pilot work as for the main studies.

This would improve quality and consistency, promote transparency, and facilitate understanding. The availability of a larger number of high-quality examples would enable further improvements for the conduct of such studies and provide valuable results for other workers in this field.

The Consolidated Standards of Reporting Trials (CONSORT) statement is a guideline designed to improve the transparency and quality of the reporting of randomized controlled trials (RCTs) [17]. Adherence to CONSORT recommendations for RCTs is already recommended by many journals in their instructions to authors. Weaknesses in the reporting and conduct of pilot trials have been identified [7, 10]. The CONSORT guideline has therefore recently been extended to provide recommendations and a checklist for randomized pilot and feasibility trials conducted before the main RCTs [12]. Although the extension covers different terminologies (pilot, feasibility, trial, study), it does not apply to internal pilot studies that are built into the design of a main trial, or to non-randomized pilot and feasibility studies. However, much of what is presented could be adapted to apply to these types of pilot or feasibility studies or similar types of trial.

Qualitative research is often used alongside other methods to assess feasibility and there are variations in how this is used and how it is reported. Some advice and references to other guidelines for qualitative studies are provided [12].

Adherence to guidelines would be helpful for other researchers designing studies in the future and would facilitate the evaluation of manuscripts by peer reviewers and editors.

The growing number of studies described as feasibility or pilot studies is reflected by the recent introduction of new journals specifically covering this important area of work.

### Recommendation 3: Clearly defining the different objectives of the pilot study in reports and publications in PHIR

There are many types of pilot studies with many different objectives.

The objectives and research questions should drive the methods used and they need to be clearly stated for the purpose of evaluation of the intervention and for process evaluation. It is also a sine qua non condition to encourage, advocate, and train in the use of pilot studies in PHIR.

### Recommendation 4: Encouraging complete pilot studies for de novo research-designed complex interventions

As detailed, pilot studies could prepare for both the evaluation and the intervention.

In addition to testing the feasibility of the trial, the pilot study can be used to test the intervention’s activity or strategy, to validate components and intervention theory, to test a way of operating/administrating an intervention, to explore how intervention protocols are followed by practitioners and clients, and to assess viability.

They serve to improve the intervention which will be delivered and hence improve the results expected by exploring how the deliverable works.

But this approach has to be structured and supported. It could be interesting to design and promote a guideline explaining the different aspects to be studied, how and in which stages of the intervention and evaluation development, and encouraging funders to support pilot studies.

### Recommendation 5: Integrating evaluability assessments as pilot studies for field-designed programs in PHIR

As a field-designed program has not initially been designed for the purpose of knowledge generation, it is important to assess to what extent suitable and reliable data can be extracted when the program is run. This is the purpose of evaluability assessment.

The evaluability assessment goes further than merely providing information on whether a program can be evaluated or not. It is used to describe the objectives, logic, and activities of the program with the aim of investigating its credibility, feasibility, sustainability, and acceptability. It ensures that there is an interest in evaluating this program.

## Conclusions

Pilot studies play an important role in intervention and PHIR. There are many types of pilot studies with many different objectives. They are of value in understanding the feasibility of the intervention and optimizing its design and evaluation. The questions to be addressed should drive the approach and methods. When considering the place of pilot studies in process evaluation for PHIR there are many challenges, including terminology. Although pilot studies fit well with the biomedical research model, for PHIR this model and approach are not always appropriate. The consensus was that a pilot study is generally appropriate for a de-novo research-designed program whereas evaluability assessment may be more appropriate for real-life programs.

The increasing use of pilot studies in complex interventions and the sharing of information and experience across many different disciplines, through better reporting, should provide a better understanding of the place and value of pilot studies in PHIR. On the one hand, pilot studies increase the appropriateness of the investigation methods (i.e., quality of research methods). On the other hand, they allow intervention mapping (feasibility, components, contextual factors, fit for real conditions), which is needed for the knowledge transfer process. These two conditions for evidence-based decision-making and pilot studies could thus facilitate evidence-based decisions in public health policy.

## Data Availability

Not applicable.
